# The Prevalence and Impact of Vaccination Programs: Differences between Nursing Homes and Assisted Livings

**DOI:** 10.1093/geroni/igaf122.2698

**Published:** 2025-12-31

**Authors:** Kennedy Berner, Patrick Mese, John Bowblis

**Affiliations:** Miami University, Oxford, Ohio, United States; Miami University, Oxford, Ohio, United States; Miami University, Oxford, Ohio, United States

## Abstract

Vaccinations are a critical component in public health for preventing infectious disease outbreaks, particularly in high-risk populations living in congregate settings. Vaccinations are an important tool to protect this population; however, we have limited knowledge about vaccination programs in nursing homes (NH) and assisted livings (AL). This study utilized a unique survey of all NHs and ALs in Ohio (response rate > 90%) – the 2023 Ohio Biennial Survey of Long-Term Care Facilities – to understand the prevalence of vaccination programs and whether having a vaccination program resulted in fewer hospitalizations and outbreaks that led to the suspension of new admissions. Vaccination programs were more prevalent in NHs than ALs for flu (100% vs. 98.8%), COVID-19 (99.5% vs. 93.3%), RSV (76.7% vs. 50.5%), pneumococcal (99.6% vs. 85.0%), Tdap (58.6% vs. 37.7%), shingles (72.3% vs. 46.7%), and hepatitis B (80.9% vs. 55.8%). NHs and ALs with a vaccination program reported that residents were less likely to be hospitalized for that condition. Fewer than 5 NHs and ALs (< 0.5%) suspended admissions due to flu or RSV outbreaks, however, 9.6% of NHs and 6.9% of ALs suspended admissions due to COVID-19. Despite facing similar risks from vaccine-preventable illnesses, ALs were less likely than NHs to have vaccination programs. These programs reduce the risk of hospitalization and disruption of admissions. Furthermore, most vaccinations programs focus on respiratory diseases. Providers and policymakers may want to place greater emphasis on having programs for all vaccine preventable illnesses.

